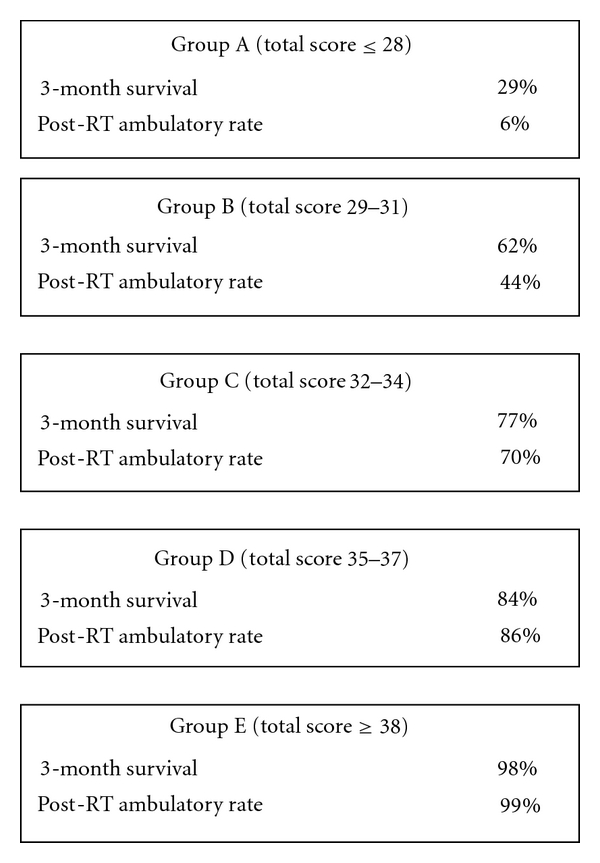# A Systematic and Evidence-Based Approach to the Management of Vertebral Metastasis

**DOI:** 10.5402/2011/719715

**Published:** 2011-08-02

**Authors:** Richard L. S. Jennelle, Vani Vijayakumar, Srinivasan Vijayakumar

**Affiliations:** ^1^Department of Radiation Oncology, University of Mississippi Medical Center, 2500 N. State Street, Jackson, MS 39216, USA; ^2^Department of Nuclear Medicine, University of Mississippi Medical Center, 2500 N. State Street, Jackson, MS 39216, USA

## Abstract

Diagnosis and management of vertebral metastasis requires a systematic approach to patient identification as well as selection of appropriate therapy. Rapid identification and prompt intervention in the treatment of malignant epidural spinal cord compression (MESCC) is key to maintaining quality of life. This paper provides a series of tools as well as guidance in selecting effective and evidence-based therapy individualized to the specific patient.

## 1. Introduction

Managing vertebral metastasis is a common problem in modern oncology. It is also a great deal more complicated than it would first appear. The problem spans the extremes of modern oncologic management from simple palliation to the newly recognized situation of “oligometastasis” which implies the potential for durable remission. The last twenty years have seen the introduction of advanced imaging which has greatly expanded our ability to detect and quantify this disease noninvasively. The last ten years introduced a systematic and ever-increasing body of knowledge, as well as techniques, which have increased our ability to offer meaningful intervention. The purpose of this paper is to outline a logical and evidence-based approach to the diagnosis and management of vertebral metastasis. Choosing wisely among the many options will provide optimum outcome for patients in a cost-effective manner. 

Among the most potentially devastating complications of metastatic cancer is malignant epidural spinal cord compression (MESCC), and so, that seems a logical place to start. MESCC is an oncologic emergency that lies hidden among the forest of simple vertebral metastasis. Prompt recognition and appropriate intervention has a profound impact on patient quality of life as well as overall survival. Maintaining optimal sensory and motor function is imperative. As such, clinicians need to be aware of this entity and have a logical approach to its evaluation and management. Any patient with a history of malignant disease who presents with back pain or any focal neurologic deficit should be evaluated urgently for the possibility of MESCC.

## 2. Diagnostic Evaluation of MESCC

As always, a careful history and physical examination is indicated. Unfortunately, the history and physical examination does not appear to be sufficiently sensitive to stratify patients who need definitive imaging evaluation [1, 2]. A history of cancer, especially when it is known or suspected to be metastatic, should raise the possibility of MESCC especially when it is combined with any focal neurologic deficit. Pain, although common, is not a requirement in the setting of MESCC [3]. Pain can be localized to the spine or it may be radicular in nature. Frequently, patients may be on analgesics which could mask the pain as well as contribute to constipation or urinary hesitancy. Both constipation and urinary hesitancy can be signs of MESCC as well.

Focal findings of weakness should always raise the concern of MESCC in a patient with cancer, but like pain, it is not a requirement of the diagnosis [1]. A careful neurologic exam may often assist in localizing a level of the cord that is clinically suspicious for cord compression. Although the history and physical examination are important, they cannot reliably identify those patients who are not at risk of MESCC [1]. Definitive imaging obtained in an urgent manner is required.

The modern gold standard for the imaging evaluation of MESCC is MRI of the entire spine [4]. Although simpler and less expensive imaging has been advocated in the past prior to requesting an MRI, these imaging tests have not demonstrated any significant ability to abrogate the need for MRI. They only delay the definitive imaging study and, so, they should be avoided when clinical suspicion of MESCC is raised. However, once MESCC has been ruled out, further imaging and other clinical parameters can have a significant impact on patient management and should be integrated into the evaluation and management process [3]. 

In the rare situation in which MRI is either not available or not safe for a particular patient, the other acceptable imaging study is myelography. Although some authors consider this potentially more sensitive than MRI, the difference in detection is likely confined to subtle and early lesions. This potential advantage is offset by the invasive nature of the study. When performed, the initial study is usually a lumbar myelogram. In the situation where a complete block to CSF flow occurs, it is necessary to supplement the study with a cervical myelogram to accurately assess the extent of involvement as up to 30% of MESCC can have multiple levels involved [4]. 

## 3. Management of MESCC

Once MESCC has been diagnosed, prompt management of the disease is necessary for optimal patient outcome. The first step is to initiate management with steroids in patients who have focal neurologic deficit [5]. Optimal steroid management is a subject of significant debate. Although there is some evidence in support of high dose steroids [6], there is also a concomitant increase in the observed serious consequences of their use. Since many of these patients will come to the attention of spine surgeons, it would be prudent to understand and follow an institutional protocol for steroid management that has been developed in consultation with the surgical service involved.

Traditionally, MESCC has been a disease managed with radiotherapy. More recently, a multi-institution phase III trial by Patchel et al. has established a role for surgery in the management of this disease [7]. It is important to bear in mind that the patients in this study all had MESCC which was defined, at a minimum, by displacement of the cord by mass within the spinal canal. It is also important to keep in mind that the surgery performed was a maximal safe debulking of the tumor and not a simple posterior laminectomy. This study was halted early when an interim evaluation met the predetermined stopping criteria. Patients who received surgery as a component of their therapy had both a prolongation of survival as well as preservation of ability to walk. In this study, surgery was combined with postoperative radiotherapy when the patients had adequately healed. Surgical intervention in this patient population is not only effective at preserving ambulatory status, but also a cost-effective treatment [8].

**Figure 1:**
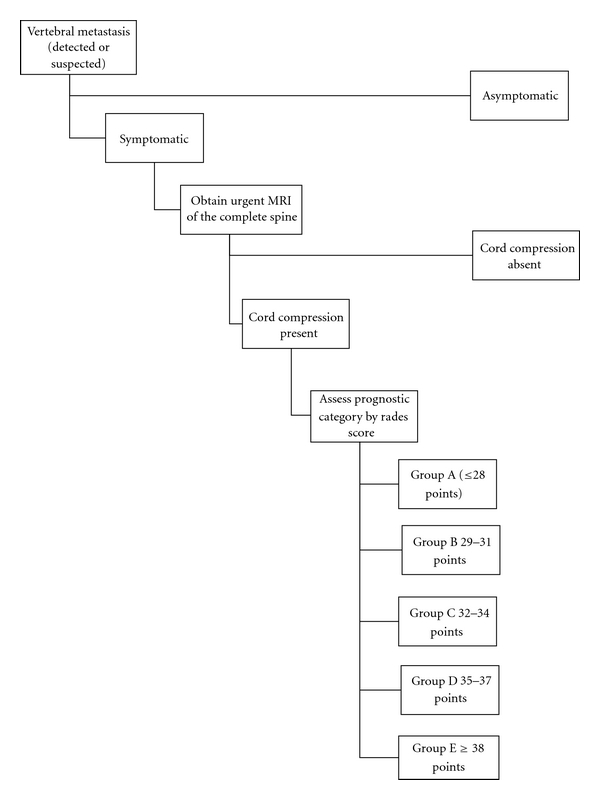


**Figure 2:**
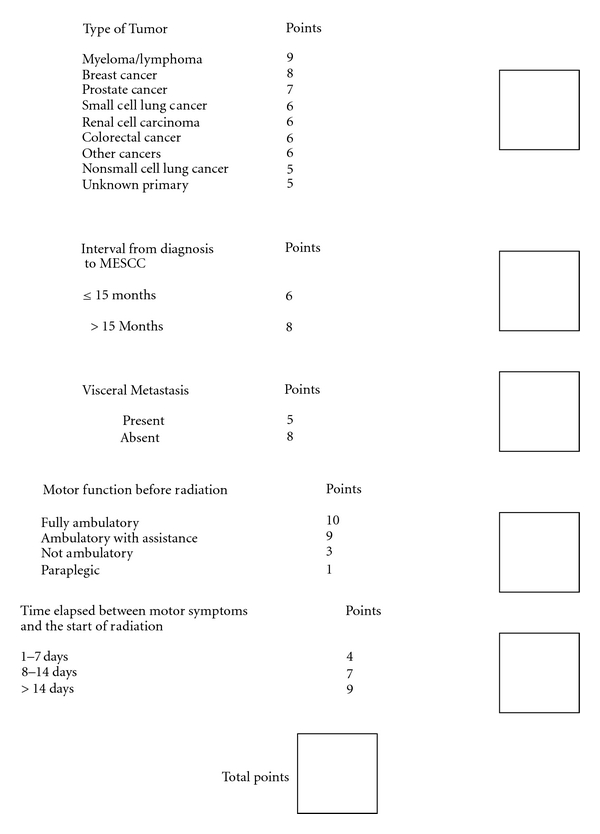


One of the main disadvantages to the surgical treatment of MESCC as advocated by Patchel is the extent of surgery many patients require. It is difficult to justify the risks of surgical treatment if the patient does not have a significant life expectancy. Until recently, it has been difficult to separate out disparate classes of patients with MESCC especially with respect to life expectancy. In 2008, Rades et al. published the results of an international retrospective review of over 2000 patients treated for MESCC with radiotherapy only [9]. Based upon this information, they were able to construct a scoring system that accurately predicts both patient longevity as well as the ability of radiation to preserve ambulatory status. This scoring system has subsequently been validated on a separate patient population [10]. Rades et al.'s work amplifies the pioneering work of Patchel by identifying patients who are likely to be candidates for surgical intervention. Patients in Group A have such limited life expectancy that rapid palliative measures make the most sense, while patients in Group E are quite well served by the use of radiation alone. Groups B, C, and D represent those patients whose life expectancy is long enough that ambulation is a serious concern and radiation alone does not adequately address this (see Figures 1, 2, and 3).

The nonsurgical management of MESCC, as demonstrated in the article by Rades et al. [9], can be effective in the long-term management of this disease. Standard radiotherapy can be effective in the palliation of both pain and neurologic dysfunction, and there is no clearly superior regimen in addressing the acute symptoms.

Until recently, it has been unclear whether neurologic function, specifically ambulation, is adequately addressed with a single-fraction treatment course. Recent prospective randomized trials have demonstrated that, in fact, single-fraction radiation is equally as effective as more protracted courses with regard to ambulation [11–13]. There is, however, a suggestion that patients who have a longer predicted survival may have less progression or recurrence of MESCC with courses of 3 Gy × 10 or longer [12]. So, for patients whose Rades score places them in Group E, a more protracted course or more aggressive therapy might be indicated.

The radiotherapeutic options continue to increase with the recent introduction of stereotactic body radiotherapy (SBRT) [14]. This new technique builds upon the long history of MESCC as a disease amenable to radiation treatment. Often, SBRT is delivered as a single fraction of radiation as well, but the dose is substantially higher than 8 Gy and would be myelopathic if delivered to the spinal cord [15]. Because of the potential danger involved, this technique must be delivered in a meticulous manner in a program which meets the quality assurance process as outlined in the ASTRO/ACR guidelines [16]. When done appropriately, 80%–90% permanent local control can be achieved without myelopathic injury even in patients with metastases known to be resistant to conventional doses of radiation [15]. This form of radiation treatment is substantially more expensive than conventional radiation and can only be delivered at select centers. The extent to which SBRT plays a role in the management of MESCC is still undefined.

## 4. Followup of Patients Treated for MESCC

Once patients have been successfully managed for MESCC, they are at increased risk for developing either a relapse of MESCC or recurrence of the process at a different site. This is especially true for patients who suffer from hormone-resistant prostate cancer. Risk stratification of patients in this situation was published by investigators at the Princess Margaret hospital, and based upon their findings, they make some suggestions on repeat imaging [3]. For patients with rapidly progressing disease, imaging as frequently as every 4 months might be necessary. More slowly progressive disease could be followed at yearly intervals. These findings have been corroborated by others [17, 18]. It remains to be seen if similar recommendations can be made for other slowly progressive systemic cancers. It appears prudent that orderly followup should be done in order to intercede before the situation becomes an emergency with all the attendant risks associated.

### 4.1. Palliation of Symptomatic Vertebral Metastasis Uncomplicated by MESCC

Pain is an emergency and needs to be treated as such. Once the evaluation for MESCC has been initiated, it is imperative that adequate analgesia be instituted. Pain is an emergency, but it is not a radiotherapeutic emergency. Proper and safe treatment planning for patients with vertebral metastasis requires that the patient be able to cooperate. Cooperation may be impossible if the patient is in acute pain. Either the managing clinician or an appropriately skilled consultant should be certain that proper pharmacologic management is instituted. It is often helpful to have a rational and evidence-based approach to palliation. Many professional societies have promulgated guidelines [19] regarding relief of pain, but it still remains a pernicious problem. 

Even in a situation where radiotherapy is immediately available, the average latency from delivery of treatment to initiation of pain relief is approximately 2 weeks, and maximum pain relief may not occur for a month or longer. Adequate pharmacologic relief of pain must be provided at the time of diagnosis and adequate arrangement made for followup and titration of the medications as needed.

Probably the most overstudied problem in radiotherapy is the palliation of pain from bone metastasis. Many high quality prospective randomized trials have been performed with many different radiotherapy regimens. No protracted treatment course has ever been found superior to single-fraction (8 Gy) radiotherapy. This is further reinforced by the meta-analysis conducted by the Cochran Collaborative [20]. The risk of developing MESCC or pathologic fracture is not increased when comparing single fraction radiation to a fractionated course. This was demonstrated in the British Bone Pain Working Trial, which was designed with that endpoint in mind [21]. It is interesting to note that multiple trials have confirmed, although there is no difference in palliation of pain, that there is an increased use of repeat treatment with the single-fraction regimens. Most feel retreatment represents greater physician and patient acceptance of the convenience of the single fraction regimen and willingness to pursue further treatment for greater relief.

## 5. Summary

Vertebral metastasis represents a spectrum of clinical significance that should be systematically approached in order to secure the optimal outcome for each patient. Rapid recognition of the potentially devastating situation of MESCC followed by prompt, evidence-based intervention results in improved outcome for patients. Adequate pain relief initiated by pharmacologic intervention and followed by focal therapy minimizes suffering. Recognizing the special situations in which surgical intervention or more advanced radiotherapeutic techniques apply can have extraordinary impact on select subsets of the patient population.

## Figures and Tables

**Figure 3:**